# Smartphone-Based Experimental Analysis of Rainfall Effects on LTE Signal Indicators

**DOI:** 10.3390/s25020375

**Published:** 2025-01-10

**Authors:** Yiyi Xu, Kai Wu, J. Andrew Zhang, Zhongqin Wang, Beeshanga A. Jayawickrama, Y. Jay Guo

**Affiliations:** Global Big Data Technologies Centre (GBDTC), University of Technology Sydney (UTS), Sydney, NSW 2007, Australia

**Keywords:** rainfall, LTE signal, signal strength indicator, signal measurements

## Abstract

This work investigates the impact of rainfall on cellular communication links, leveraging smartphone-collected measurements. While existing studies primarily focus on line-of-sight (LoS) microwave propagation environments, this work explores the impact of rainfall on typical signal metrics over cellular links when the LoS path is not guaranteed. We examine both small-scale and large-scale variations in signal measurements across dry and rainy days, considering diverse locations and time windows. Through statistical and spectral analysis of a large dataset, we uncover novel insights into how rainfall influences cellular communication links. Specifically, we observe a consistent daily fluctuation pattern in key cellular metrics, such as the reference signal received quality. Additionally, spectral features of key mobile metrics show noticeable changes during rainfall events. These findings, consistent across three distinct locations, highlight the significant impact of rainfall on everyday cellular links. They also suggest that the widely available by-product signals from mobile phones could be leveraged for innovative rainfall-sensing applications.

## 1. Introduction

Environmental changes, particularly meteorological disasters, significantly impact human safety, infrastructure, and ecosystems. Timely and accurate monitoring is essential for effective disaster management and damage mitigation. Environmental sensing plays a key role in this process, covering areas such as pollution, barometric pressure, and water level monitoring. Among these, rainfall monitoring is crucial for minimizing rainstorm damage and supporting agriculture by providing actionable insights.

Traditional rainfall detection methods rely on physical devices to collect and calculate precipitation rates but often require frequent maintenance and calibration, limiting their reliability and scalability [1,2,3]. In recent years, leveraging ubiquitous communication links for rainfall monitoring has emerged as a promising research area. The variation in atmospheric conditions [4] can impact signal characteristics [5]. Raindrops, as a type of atmospheric particles, attenuate signal strength during signal propagation. The relationship between signal attenuation *A* (in dB) and rainfall is expressed as $A=aR^{b}L$, where *a* and *b* are related to the signal frequency, polarization state, and the drop size distribution of the rain; *R* is the rainfall rate (in mm/h); and *L* is the path length (in km) [6]. The International Telecommunication Union provides standardized parameter values to calculate rainfall attenuation under different wireless configurations [7]. This attenuation effect allows base stations (BSs) to function as virtual sensors for rainfall monitoring [8]. Exploiting the interaction between wireless signals and environmental factors provides a cost-effective and sustainable approach, as it leverages existing infrastructure. The viability of commercial microwave links (CMLs) for extended, widespread operational rainfall monitoring purposes has been demonstrated [9]. Both model-based and data-driven methods for rainfall estimation using CML data have been comprehensively reviewed [10]. Furthermore, studies have highlighted the accuracy, algorithms, and results of precipitation monitoring based on CML signal levels [11,12,13].

However, acquiring CML data from BSs is often challenging. In many cases, access to such data requires collaboration with local network operators. Moreover, the widespread deployment of underground fiber-optic cables for inter-tower communication poses additional challenges for obtaining CML data, even for network operators [14]. In contrast, wireless communication links between user terminals and BSs are much more available nowadays. Key signal indicators, such as reference signal received power (RSRP), received signal strength indicator (RSSI), and reference signal received quality (RSRQ), are readily available on mobile devices, as defined by cellular standards. Compared with CML data, user terminal data can primarily provide measurements of the received signal. Research on leveraging the above signal data collected from user devices for weather sensing is significantly less explored. Investigations of the effect of rainfall on the above signal data collected by user terminals are summarized in Table 1. For instance, the daily average RSSI values of 2G links decrease during rainfall events [15], while another study observed that average RSSI values vary with rain intensity [16]. However, the predicted RSSI degradation during rain is not always evident in practice, with occasional instances showing no significant change [15]. An inverse relationship between rainfall and RSRP is observed under a line-of-sight (LoS) scenario in the 3.5 GHz citizens broadband radio service (CBRS) [17]. Most studies relying on smartphone-measured signals require locations with direct sight paths between BSs and data collection points to ensure LoS conditions, limiting their applicability for broader rainfall-sensing implementations.

In this work, we investigate the impact of rainfall on key mobile signal metrics under realistic non-line-of-sight (NLoS) scenarios, which are more common in urban and suburban environments. At the current stage, although 5G is rapidly developing, the primary network coverage in Australia remains LTE [18]. In addition, 5G network coverage in some areas might still be patchy and inconsistent. To ensure the generalizability and reliability of our research, we have chosen to conduct this phase of the study under the LTE network coverage that is more uniformly established across the region. We conduct measurements of LTE signal indicators (RSRP, RSSI, RSRQ, and SNR) at three different locations under NLoS conditions. Both small-scale and large-scale variations are analyzed during dry and rainy days. Our findings reveal that daily patterns in LTE signal indicators are evident on dry days. By employing spectrum analysis techniques, we find that rainy days display increased power at lower frequencies for both RSSI and SNR measurements compared to dry days.

The remainder of this paper is organized as follows: Section 2 introduces the physical-layer basics and performance metrics of LTE BSs. Section 3 details the experimental setup and data collection process, followed by data pre-processing in Section 4. The data-driven results are presented in Section 5, with a comparison to previous work and discussion on future directions in Section 6. Finally, Section 7 concludes the findings.

**Table 1:** Research investigating the impact of rainfall on signal data collected by the user terminal.

Reference	Signal Frequency	Distance Between BS and User Terminal	Signal Features	Insights
[19]	3G (2100 MHz) WiFi (2.4 GHz, 5 GHz)	a few meters (LoS)	received signal level (RSL)	Measurements with 5 GHz WiFi signals have more potential to detect regional heavy rainfall.
[20]	GSM (1.8 GHz)	400 m	received signal strength	Traditional rain attenuation models have largely discounted the impact of precipitation on GSM signals.
[21]	GSM (800 MHz–3 GHz)GPS	-	received signal strength	The decision tree model infers there is rain when signal strength is quite low.
[15]	2G	-	RSSI	The average of RSSI in the rain is lower than the average in the whole day, but such decrease is not as significant as expected from the theoretical analysis.
[22]	LTE	200 m	received signal strength	The hourly average received signal level in rain is lower than that in no-rain cases.
[16]	LTE	200 m (LoS)	RSL	The mean and variance of the probability density distributions in different rain conditions are different but not enough to distinguish different rain intensities.
[23]	LTE	-	RSRP, RSL, Signal-to-Noise Ratio (SNR), RSRQ, Cell ID	The separation of different rainfall classes is not linear on signal data and cell selection-related parameters.
[14]	LTE	-	proportions of RSRP	Changes to the proportion of the weak coverage samples could be impacted by the propagation losses including rainfall influence attenuation.
[24]	lower than 10 GHz	-	RXL, RSRP, RSRQ, SNR, quality	There is a strong correlation between RXL and rainfall level (−0.78).
[25]	LTE (2630 MHz)	228 m (LoS)	RSSI	The precipitation induces a decline in signal power, and the signal power does not increase immediately after the rain stops.
[17]	CBRS private LTE network (3560 MHz, 3640 MHz)	distance not mentioned (LoS)	RSRP	There is an inverse relationship between rainfall and hourly sampled RSRP upon the least squares regression analysis based on signal data during two rain events.

## 2. Basics of BS Information and LTE Performance Metrics

This section introduces the fundamentals of LTE physical-layer signals, which are crucial for identifying the BS from which the signal is transmitted. Additionally, we will cover the key performance metrics that assess the quality and strength of the received LTE signal, which will be utilized in the rainfall sensing study presented in this work.

### 2.1. BS-Related LTE Physical-Layer Basics

A public land mobile network (PLMN) [26] is a network that uses Earth-based BSs to provide mobile communication services to terrestrial subscribers. It is identified by the combination of a mobile country code (MCC), which represents a country or geographic area, and a mobile network code (MNC), which is unique to each operator. The PLMN’s structure and communication framework are critical for identifying and utilizing the signal data transmitted by the LTE network. The LTE network is organized into tracking areas to optimize mobility management and resource allocation. A tracking area is a logical region that allows a user to move within it without notifying the network. Each tracking area contains multiple BSs, which cooperate to manage the user equipment within the area. The tracking area code (TAC) uniquely identifies each tracking area, and understanding its distribution is important for interpreting signal data from different locations, particularly when assessing the impact of rainfall on signal quality.

The evolved Node B (eNodeB) is the base station in the LTE network, each of which is assigned a unique eNodeB ID. A single eNodeB typically covers several cells, and each cell is identified by a cell ID. The physical Cell ID (PCI) is used as a physical-layer identifier for a cell, enabling user equipment to decode the transmitted data. The knowledge of which eNodeB and cell a user terminal is connected to is crucial for correlating changes in signal quality to environmental factors (e.g., rainfall). When a smartphone moves between cells, the PCI value changes, and these transitions can be tracked to detect variations in signal parameters caused by weather conditions.

### 2.2. LTE Performance Metrics

After identifying a cell, the user terminal calculates performance metrics to facilitate communications. A few signal strength and quality parameters for an LTE network, as calculated in a mobile terminal, are briefly illustrated below. These metrics will be used in this work to investigate the impact of rainfall on LTE signal quality.

(1) RSRP indicates the power level of the LTE reference signals. It is generally calculated by averaging the power of reference signals in a downlink frame [27]. In a frequency-division duplexing LTE system, one downlink frame consists of ten subframes, and each subframe contains two time slots with seven symbols. The cell-specific reference signals are placed in symbols indexed at 0, 4, 7, and 11 in each subframe. These reference signals are distributed across subcarriers, with every sixth subcarrier carrying a reference signal. The placement of these signals depends on the physical cell ID.
(2) RSSI measures the total average signal power, including both reference symbols and other co-channel interferences. Thus, RSSI is the sum of RSRP and any additional interference present in the channel. While RSRP focuses on the reference signals, RSSI provides a broader measure of the overall received signal strength.
(3) RSRQ reflects the quality of the reference signals. It jointly considers RSRP, RSSI, and the number of resource blocks (N) over the same bandwidth for measuring RSRP and RSSI. The relationship between these parameters is given by [27]
  (1) $$\begin{array}{cc} \; & \mathrm{RSRQ}=(N\times \mathrm{RSRP})/\mathrm{RSSI}\\ \; & \mathrm{RSRQ}\left(\mathrm{dB}\right)=\mathrm{RSRP}\left(\mathrm{dBm}\right)-\mathrm{RSSI}\left(\mathrm{dBm}\right)+10\times \log_{10}N \end{array}$$
  Specifically, RSRP and RSRQ are calculated by LTE user terminals and reported to the BS, acting as the criteria for the cell selection and handover algorithm [28].
(4) SNR is a metric used to indicate the quality of the received signal. It is defined as the linear average over the power contribution of the resource elements carrying cell-specific reference signals divided by the noise power. The noise power could be estimated by the sum of the average linear power values of signals transmitted by non-target BS over the OFDM symbols carrying cell-specific reference signals [29]. A higher SNR indicates better signal quality, while a lower SNR suggests greater interference or attenuation.

In this work, these LTE performance metrics, including RSRP, RSSI, RSRQ, and SNR, will be analyzed to determine how rainfall affects signal quality and whether they can be used as reliable indicators for rainfall sensing.

## 3. Experimental Setup and Data Collection

This section outlines the methodologies and procedures implemented in our study to ensure robust data collection and analysis. The experimental setup is designed to systematically investigate the relationship between LTE signal performance and environmental conditions. We will detail the equipment used, the data collection methods, and the selected measurement locations for our experiments.

### 3.1. Experimental Setup

**Measurement Locations and Setup.** Three different measurement locations were selected for data collection to investigate the impacts of deployment environments on mobile signal-based rainfall sensing. For each location, the signal data are captured by smartphones. The mobile terminal used in our experiments is the Nokia X20 [30]. To mitigate the effects of phone motion on signal measurements, the smartphone is kept stationary at a fixed location during the data collection process. The same device is used for the entire duration of the experiment to maintain consistency in the collected data.

**Figure 1:**
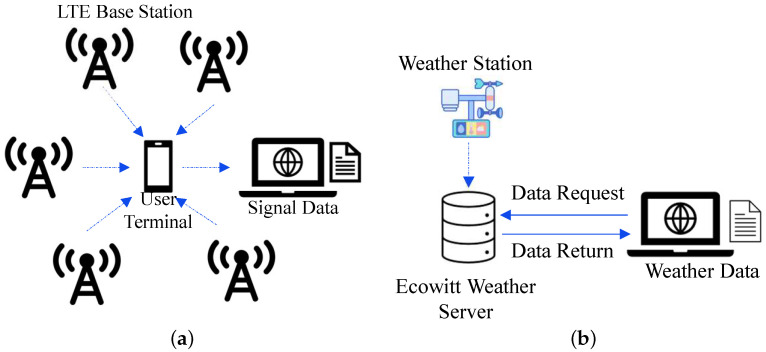
Experiment setup: (**a**) Signal data collection and transfer. (**b**) Weather data collection and transfer.

**Signal Parameter Collection.** Several mobile applications are available for measuring LTE power metrics. Among them, G-Mon is the most widely used application for capturing signal indicators [16,23,24]. G-MoN Pro [31] is used to achieve the extraction and saving of signal measurements such as RSRP, RSSI, RSRQ, SNR, and other LTE metrics from the serving BS since G-Mon is more suitable for the relatively old Andriod version. While signal parameters from neighboring cells can be viewed on the screen, they cannot be saved to files. The mobile application is configured to store each set of signal metrics along with a timestamp at fixed intervals as Figure 1a, ensuring that the signal data are closely aligned with the weather data.

**Ground Truth.** Ground truth weather data are obtained from publicly accessible weather stations shared on the Ecowitt platform [32]. Thanks to the widespread deployment of Ecowitt Internet of Things (IoT) weather stations, we are able to locate stations near our experimental sites. This allowed us to acquire rainfall data for each mobile terminal used in our study, with a time resolution as fine as 5 min, as provided by Ecowitt’s weather data shown in Figure 1b.

### 3.2. Data Collection

**Figure 2:**
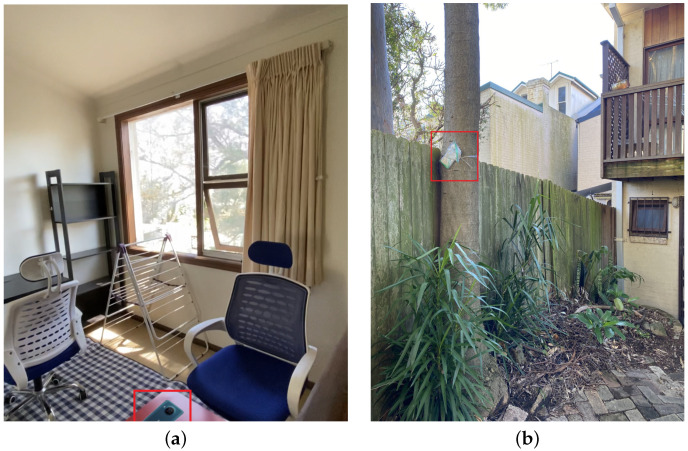
Signal data collection locations: (**a**) Signal data collection location 1. (**b**) Signal data collection location 2.

The data collection is carried out across three locations in Sydney, Australia, each presenting distinct propagation environments. The experiment is conducted during the frequent rainfall season from November 2023 to March 2024 [33]. Table 2 summarizes the data collection conditions and results. The first experiment’s location is on the third floor of a townhouse, where the mobile phone is put on the red desk shown in Figure 2a. The second experiment’s location is in the yard of the same townhouse, with the phone tied to a tree inside a plastic bag, as illustrated in Figure 2b. The third location is inside a typical house with the collecting mobile phone placed on a table. These diverse environments facilitate the investigations of how indoor and outdoor conditions affect mobile signal-based rainfall sensing. They also help assess the robustness of our method in varying scenarios with different mobile network configurations.

**Figure 3:**
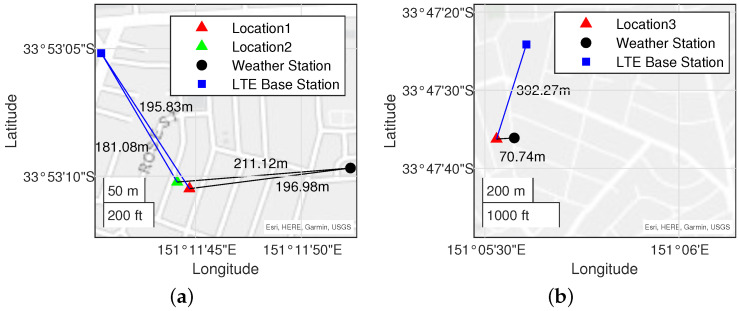
Locations for data collection: (**a**) locations for Dataset1 and Dataset2; (**b**) location for Dataset3.

The signal data collection location, weather station location, and the location of cells that the mobile terminal is most frequently connected to are shown in Figure 3. The longitude and latitude coordinates for the signal collection sites and weather stations are recorded, while the cell locations can be identified using Opencellid [34], based on the cell ID, MCC, MNC, and LAC (TAC). The distances between the weather stations and the signal data collection locations are small enough to ensure that rainfall intensity in the surrounding area is accurately captured.

**Figure 4:**
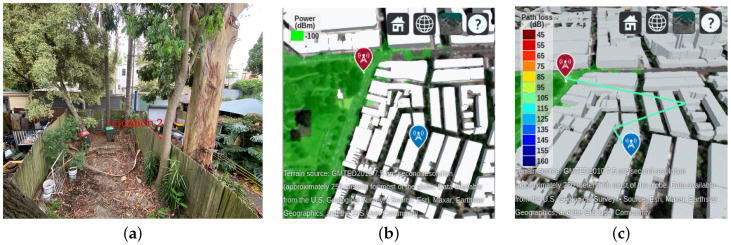
Radio propagation environment between cell and user terminal: (**a**) Cannot see the cell through visual inspection. (**b**) No LoS path was found in the radio propagation simulation. (**c**) The reflected path was found in the radio propagation simulation.

In this work, the signal data are collected where an LoS path between the cell and the smartphone is not guaranteed. This differs from most existing papers investigating the relationship between signal data and weather based on the LoS propagation path between BS and a mobile terminal. In these studies, maintaining an LoS path is generally considered, but in practice, this can be quite challenging. Here, we adopt visual inspection and ray-tracing simulation to confirm that there is no direct path between the cell and the data collection location. The experimental location 1, as noted in Table 2, is selected as an example. The surrounding environment for data collection is shown in Figure 4a. We could not see the BS. A ray-tracing simulation is performed in MATLAB R2024a with map and building files of experimental locations, as obtained from OpenStreetMap [35]. The simulation allows us to visualize the microwave propagation between the cell and the user terminal. The red marker represents the BS while the blue marker represents UE in Figure 4b,c. By configuring the simulation to ignore reflected paths, we can check for the existence of an LoS between the BS and the user terminal. As shown in Figure 4b, there is no LoS path between the BS (indicated in red) and signal data location 1 (indicated in blue). Furthermore, when the simulation is configured to account for additional reflected paths, we can observe the effects of path loss and signal degradation. Figure 4c illustrates one reflected path in experimental location 1, when the maximum number of reflections is set to two.

One of the raw signal data snippets is shown in Table 3, after removing columns with invalid values or data that are irrelevant here. The table displays the key signal power metrics, as detailed in Section 2. The column labeled ’BAND’ indicates the frequency bands used for telecommunications. For example, ‘2600 B7’ refers to the uplink frequency range of 2500–2570 MHz and the downlink frequency range of 2620–2690 MHz. The ‘BANDWIDTH’ column represents the channel bandwidth, which defines the range of frequencies allowed to pass through the channel. Finally, ‘LAT’ and ’LON’ refer to the latitude and longitude coordinates of the device location, respectively.

Specifically, several important notes are outlined below. First, non-standalone 5G is primarily used in Australia at the time of writing, which means 4G base stations may also support 5G. To maintain consistency in our study, we have restricted the data collection to 4G, as the network configuration, such as the power management strategy and the advanced technology, might differ for non-standalone 5G and LTE. In the G-Mon Pro-captured data, the ‘NR_STATE’ field can be used to filter out any 5G NR data. Second, cell handovers may occur even when the mobile terminal remains stationary at the experimental location. The ‘LOCLA_CID’ field, as shown in Table 3, can be used to categorize data entries according to the connected cell.

## 4. Signal Pre-Processing

In this section, we introduce the signal pre-processing methods employed in this work to better prepare the captured data for proceeding analysis.

The first step is cell separation. As mentioned in Section 3.2, different cells can be recorded even when the mobile terminal is stationary. Therefore, it is necessary to group the captured data entries by their associated cells and select the primary cell to which the mobile terminal is most frequently connected. As shown in Table 3, the fields ’XCI’ and ’LOCAL_CID’ are used to identify and categorize the data entries under different cells. From the data collected at the three locations described in Section 3, we can identify the main cell at each location. The results of this cell identification process are summarized in Table 4.

For data entries associated with each cell, we begin by performing data cleansing, which includes removing duplicate entries and interpolating missing values to ensure that measurements are aligned to consistent time intervals. Typically, the start and end times of the exported signal data file correspond to the moments when the control button on the screen is pressed to initiate and terminate recording. However, there are instances where the record does not end properly after the stop button is pressed. Consequently, only the measurements from the most recent file are retained for analysis. Records containing ‘nan’ values for all signal measurements are excluded.

Since mobile data and weather data have different temporal resolutions, time synchronization and alignment are necessary. The weather data resolution is limited to five-minute intervals, as provided by the open database used [32], which is significantly lower than the temporal resolution of the mobile data. To address this, interpolation is applied to match the timestamps of the two datasets. Specifically, the values from the nearest available timestamp are used to fill in any missing mobile data. The Modified Akima Cubic Hermite interpolation method [36] is then used to estimate the precipitation rate for each corresponding mobile data entry. This interpolation is implemented using the ‘makima’ method of the ‘interp1’ function in MATLAB. Additionally, any interpolated precipitation values that result in negative values are set to zero, ensuring that the data remain valid.

## 5. Data-Driven Analysis

In this section, we examine the impact of rainfall on the pre-processed mobile signal metrics. Specifically, we explore the variations in signal measurements during dry periods, analyzing both small- and large-scale fluctuations, as detailed in Section 5.1. Following this, Section 5.2 analyzes the impact of rainfall on LTE signal indicators, focusing on standard deviation and frequency.

### 5.1. Variations in Measurements During Dry Period

Before analyzing rain’s impact on mobile signals, it is important to understand how the signals behave during dry periods, without the influence of rain. To achieve this, we examine signal features within both short- and long-term windows, referred to as small- and large-scale windows, respectively. Specifically, small-scale analysis focuses on variations within an hour, while large-scale analysis investigates variations over several days.

#### 5.1.1. Small-Scale Signal Features in Dry Days

**Figure 5:**
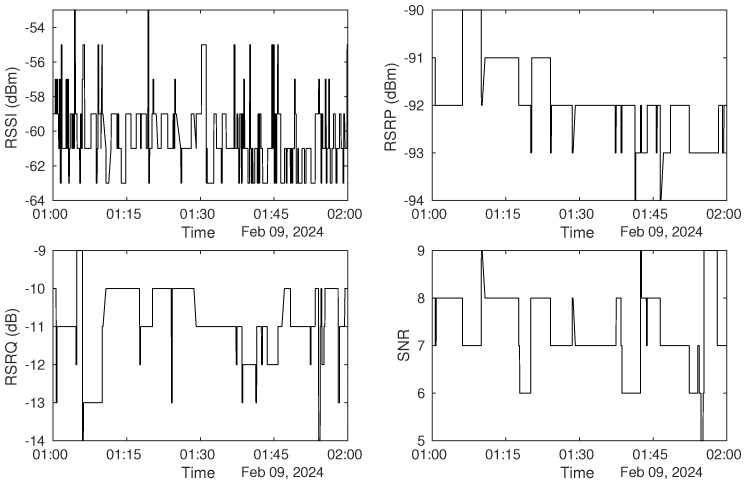
One−hour signal measurements in Dataset1.

The minimum sample interval is one second in the mobile application. A one-hour continuous time window is selected to inspect the small-scale signal variations, as shown in Figure 5.

Figure 5 shows significant fluctuations in signal measurements throughout the duration of an hour. We see consistent signal variations on the second level, which is less likely to be caused by environmental factors to be sensed. Such variations hence need to be suppressed or mitigated for accurate environmental sensing.

**Figure 6:**
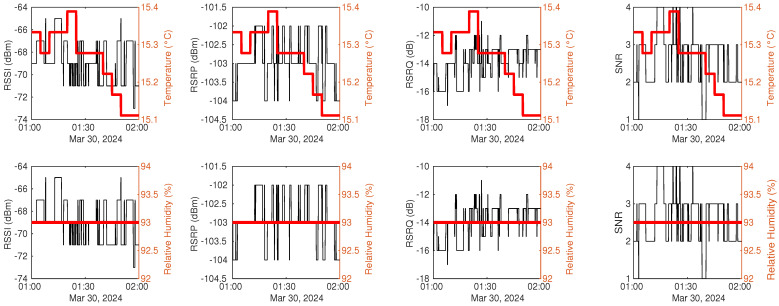
Signal measurements within an hour during the dry period in Dataset2.

Environmental factors, like temperature and humidity, might influence signal measurements. To investigate this, Dataset2, collected outdoors, is selected for analysis. At the same time as mobile signal metrics are collected, temperature and humidity measurements collected outdoors are recorded. Specifically, the time period from 1 a.m. to 2 a.m. is chosen due to less human activity near the data collection devices. Figure 6 presents one hour of signal measurement samples alongside matched temperature and relative humidity data during dry conditions. The signal measurements fluctuate much more frequently than the changes in temperature and relative humidity. Temperature only varies by 0.3 degrees while remaining constant in relative humidity. Notably, signal measurements continue to vary even when temperature and humidity remain stable from 1:20 a.m. to 1:40 a.m. This is consistent with the indoor experiment, where fluctuations in the RSSI occur without interference or disturbance [37].

**Figure 7:**
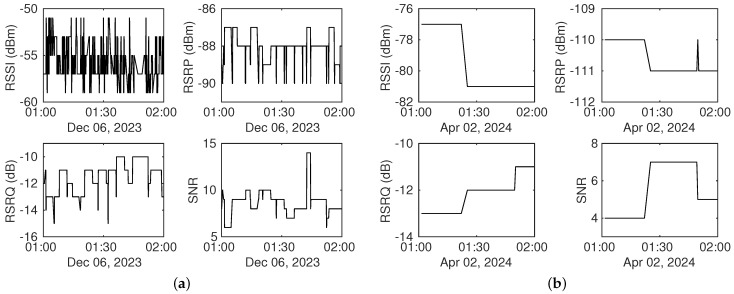
Signal measurements taken within one hour during dry period: (**a**) signal measurements in Dataset1. (**b**) signal measurements in Dataset3.

Figure 7 presents examples of signal samples from a one-hour dry period for the other two datasets. It is evident that fluctuations in RSSI measurements are more than those in RSRP measurements in Dataset1, as shown in Figure 7a, which aligns with findings from a previous study [17]. Additionally, Dataset3 exhibits fewer oscillations in Figure 7b, likely due to its larger sampling interval compared to the other two datasets.

The results illustrated above indicate that small-scale signal fluctuations exist even in dry conditions. Such fluctuations should be suppressed or mitigated to avoid interfering with rainfall sensing. Processing methods that mitigate non-weather-related variations, such as averaging measurements within a specified time window [16,23] and employing moving averages [25], have been utilized in previous studies. The moving average typically employs a time window of several minutes, using overlapping samples to compute averaged signal parameters from neighboring data points. In contrast, the averaging method utilizes non-overlapping time windows, usually with a duration of one hour. In this work, the average method among non-overlapping time windows has been employed.

#### 5.1.2. Variations at a Large Scale

To investigate large-scale variations, it is critical to mitigate small-scale fluctuations. In this study, a one-hour averaging method is employed. To further smooth the fluctuations and highlight the overall trend from a broader perspective, a low-pass filter with a normalized bandwidth of 0.3 is adopted. The lowpass filter uses a minimum-order filter with a stopband attenuation of 60 dB and compensates for the delay introduced by the filter. Using this narrower bandwidth facilitates better visualization of long-term trends in the data. It is important to note that this filtering process is intended solely for enhancing the visualization of the overall trend; the spectrum analysis is conducted on the averaged data, not the filtered data. Specifically, the Fourier transform F of *L*-length hourly average measurement sequence x(t) is utilized, and the Power Spectral Density (PSD) estimate P(f), calculated by Equation (2), is employed to analyze the distribution of power across different frequency components.

(2) $$P\left(f\right)=\frac{{2|F\left(x\left(t\right)\right)|}^{2}}{f_{s}L}$$

**Figure 8:**
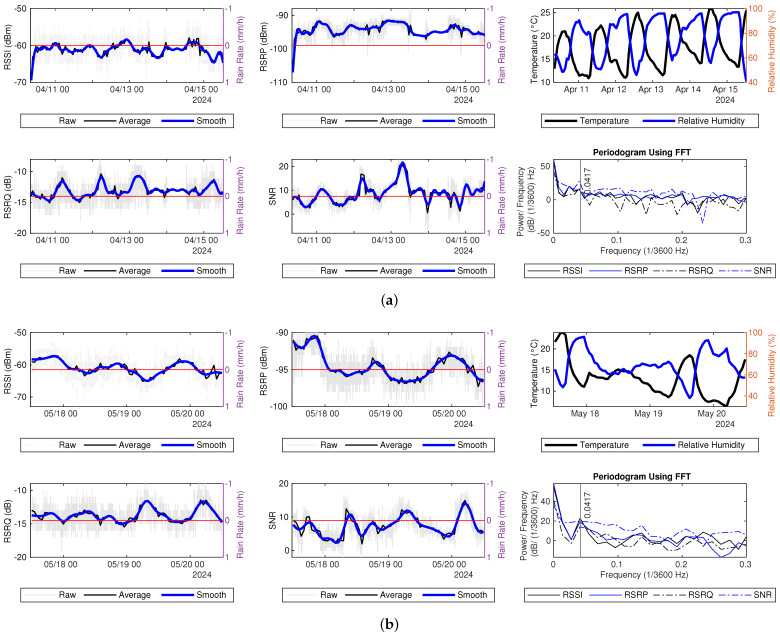
Signal measurements during dry periods in Dataset2: (**a**) 11–15 April. (**b**) 18–20 May.

The sample interval for hourly average measurements is set to one hour, corresponding to a sampling frequency $f_{s}$ of 1/3600 Hz. For simplicity, the frequency unit is defined as 1/3600 Hz. If a pattern occurs every 24 h, it should be represented as a strong power at 1/24 (×1/3600) Hz when using data with an interval of an hour. Figure 8 displays the raw signal measurements, hourly average measurements, filtered smooth data, as well as temperature and relative humidity data, along with the PSD estimate for the hourly average signal measurements.

**Figure 9:**
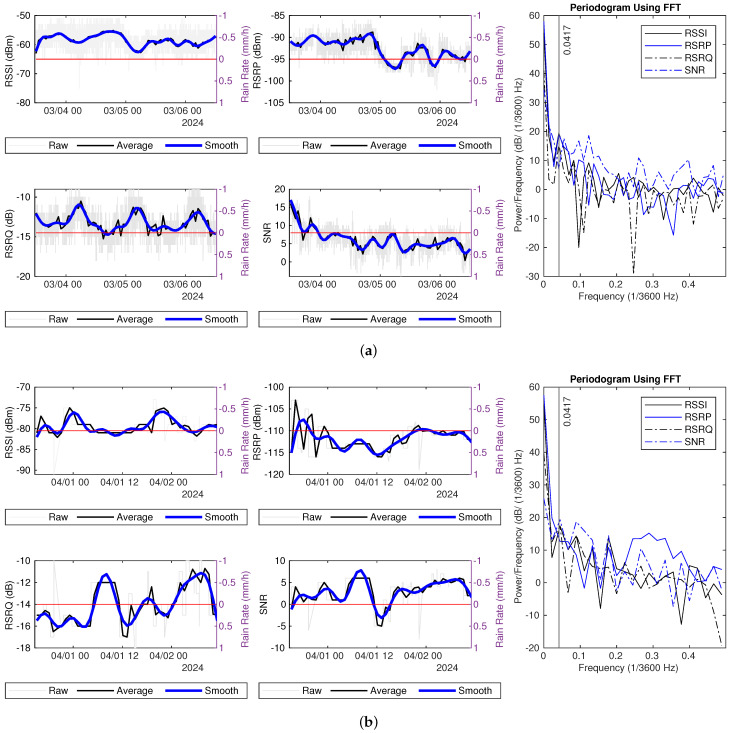
Signal measurements during dry periods: (**a**) 3–6 March in Dataset1. (**b**) 31 March–1 April in Dataset3.

Hourly averaged RSRP and RSSI values consistently drop after midnight each day and increase in the morning, except April 14th, when the values remain stable until noon, contrasting with the variations observed at other times. It is evident that the PSDs show localized peaks around 0.0417 (≈1/24) for the one-hour sample interval of RSRP, RSSI, and RSRQ. The higher power at this frequency component further confirms the presence of the daily fluctuation pattern. According to the previous study, temperature does not have a direct impact on LTE RSRP, while relative humidity has a stronger negative impact on LTE RSRP [17]. However, these daily fluctuations do not demonstrate an inverse relationship with relative humidity, suggesting that the observed daily changes may not be caused by temperature or relative humidity. A similar daily pattern can also be seen in the hourly averaged measurements and PSD for hourly average signal measurements in Datasets 1 and 3, as shown in Figure 9.

The PSDs for the four signal quality and strength metrics show a declining trend at higher frequencies. The SNR PSD estimate in Dataset1 does not have a peak at 0.0417 (×1/3600) Hz while the RSRP PSD estimate in Dataset3 does not show this peak. Since the datasets are collected at different locations and on different dates, the observed daily pattern in signal measurements is not a unique phenomenon. This may be attributed to adjustments in signal transmission power from the LTE BS or variations in network congestion at different times.

When analyzing the impact of rainfall on signal measurements at a large scale, it is important to separate the daily pattern from the effects of rainfall. To mitigate repetitive effects, the entire day is divided into four non-overlapping time windows [15]. However, based on the collected data in this study, the timing and magnitude of drops and increases in value vary from day to day. Typically, values begin to decline between 5 p.m. and 7 p.m., and then generally stop declining, starting to rise again between 5 a.m. and 7 a.m.

### 5.2. Rainfall Impact on Signal Measurements

Given the daily pattern observed in the signal measurements in Section 5.1.2, the average value is not an appropriate evaluation metric for investigating the relationship between rainfall and signal measurements. This is because any decrease in the average could result from rainfall, the daily pattern, or a combination of both. Variance remains a viable metric to explore, as rainfall intuitively induces greater diversities in signal fluctuations, expecting to vary signal variance. This section analyzes rainfall’s influence on signal measurements through standard deviation (SD). Additionally, the effects of rainfall on signal measurements are examined through spectral time series analysis.

#### 5.2.1. Standard Deviation for the Different Weather

To investigate signal features on rainy days, we begin by analyzing the SDs of RSSI, RSRP, RSRQ, and SNR. However, this requires aligning the captured data based on time.

Although the sample interval can be set manually to a fixed number, the actual sample interval may differ from this setting. This non-uniform sampling must be addressed before calculating the SD. There are two primary reasons for the variation in sample intervals. First, non-uniform sampling may arise from the device or application control. In this case, the phone maintains a connection with the same cell, but the sample interval deviates from the manual setting. Second, the switching of connected cells can interrupt data recording from the main serving cell. However, this non-uniformity constitutes a small portion of the overall dataset. For example, in Dataset1, over 94.37% of the sample intervals match the manual setting after selecting the signal data from the main serving cell. Of the remaining intervals, 5.48% of interval values deviate from the manual setting due to device control issues, while only 0.15% of deviations correspond to cell switching, resulting in intervals longer than the set value. Notably, more than 99% of the longer intervals caused by device control issues are less than one minute, and approximately 98% of the intervals resulting from cell switching are also less than one minute. In total, over 99.9% intervals are less than one minute.

**Figure 10:**
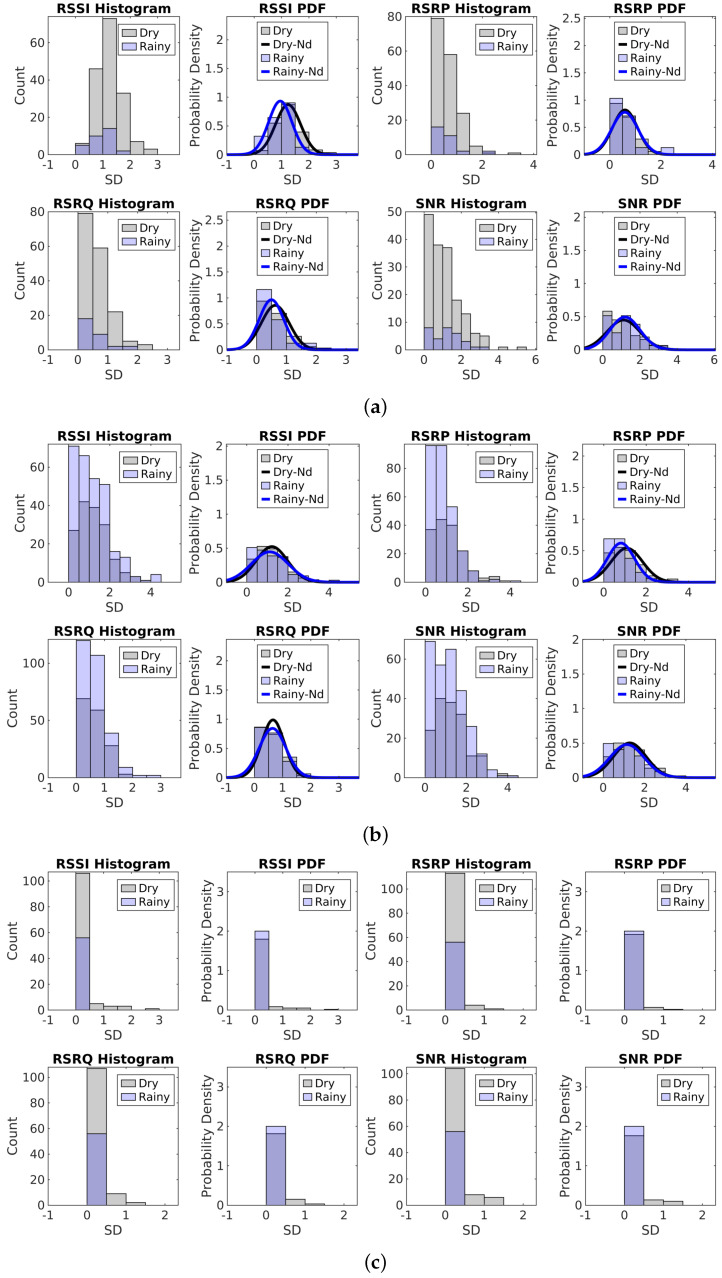
SD statistic for signal measurements: (**a**) Histograms and PDFs for the SD for 13–14 December in Dataset1. (**b**) Histograms and PDFs for the SD for 4–6 April in Dataset2. (**c**) Histograms and PDFs for the SD for 4–6 April in Dataset3.

To address the non-uniform sampling, we first calculate the average measurement for one-minute intervals, which also helps mitigate fluctuations at the seconds level. Then, we apply linear interpolation to estimate any missing values within these one-minute intervals, provided the missing duration is less than two minutes. Next, we compute the SD over a five-minute time window using these one-minute average measurements. If any missing values occur within the time window, that window is excluded from the SD calculation. The SD values are further categorized into dry and rainy classes based on the mean precipitation rate. Specifically, when the mean precipitation rate equals zero, the data are labeled as dry; otherwise, they are labeled as rainy. The SD histograms, along with the Probability Density Function (PDF) and the normal fit of the SD for dry and rainy periods during rainy days across different datasets, are presented in Figure 10.

From Figure 10, we can see that there is no significant difference in signal SD between rainy time and dry time. The distribution of the normal fit for different signal metrics under different weather conditions looks quite similar. The signal SDs during rainy periods are not larger than those during dry periods, similar to findings from an indoor experimental study [38]. Even during dry periods, there are notable fluctuations in signal measurements within an hour, as discussed in Section 5.1.1. The PDF distributions for the SD in rainy and dry conditions are almost the same across all datasets. This observation suggests the limitations of using SD alone to assess the impact of rainfall on signal measurements, especially in complex microwave propagation environments like indoor locations.

#### 5.2.2. Frequency Difference for the Different Weather

In Section 5.1, a daily variation pattern is observed. The uncertainties regarding when values change, and the extent of these changes complicate the estimation of variations and the separation of rain-induced fluctuations from those caused by other factors. Treating rainfall as an abnormal weather condition might disrupt the long-term pattern of signal measurements, potentially introducing more frequency components, which would be reflected as increased power at some frequencies in the PSD spectrum. Thus, the frequency domain has been utilized to analyze the impact of rainfall on signal measurements.

**Figure 11:**
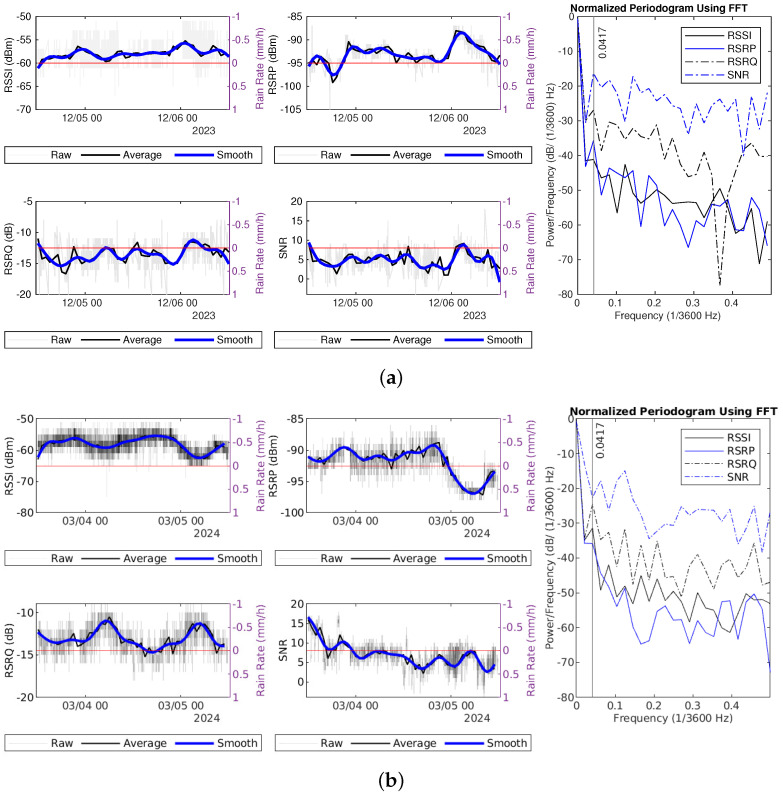
Hourly average signal measurements and NPSD spectrum during dry days: (**a**) 4–6 December. (**b**) 3–5 March.

To facilitate a comparison of the PSD during rainy and dry periods, we apply the Fourier transform to an equal number of samples, 48 (2 days), from different weather conditions. The PSD is normalized by dividing the total power across all frequencies and converting the result to decibels unit. Two examples of signal measurements for dry days, along with their normalized power spectral density (NPSD) spectra, are presented in Figure 11. The NPSDs exhibit a tailing trend; generally, higher frequencies correspond to lower power levels. This drop is more pronounced in RSSI and RSRP measurements compared to RSRQ and SNR, signifying a greater disparity in power across different frequency components.

To compare the PSD during rainy and dry periods, the NPSD for rainy days is divided by the NPSD for dry days, resulting in a subtraction between the two NPSD values expressed in decibels (dB). The difference between the NPSD values for rainy and dry conditions being greater than zero indicates more power during rain than during dry conditions at that frequency. The NPSD during rainy days and the NPSD difference between the rainy and dry periods from different datasets are shown in the figures below.

**Figure 12:**
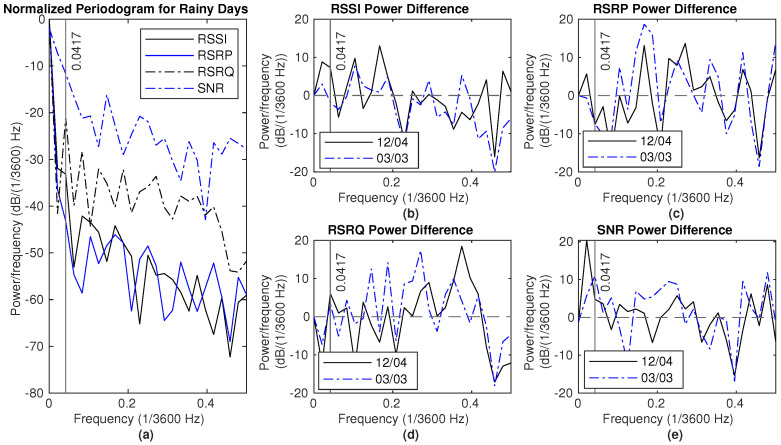
Signal measurements during rain (18–20 December): (**a**) NPSD for signal measurements. (**b**) RSSI power difference of NPSD between rainy days and dry days. (**c**) RSRP power difference of NPSD between rainy days and dry days. (**d**) RSRQ power difference of NPSD between rainy days and dry days. (**e**) SNR power difference of NPSD between rainy days and dry days.

**Figure 13:**
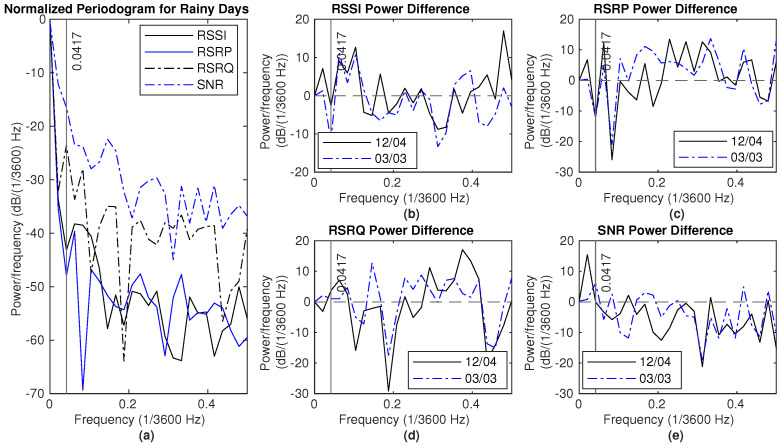
Signal measurements during rain (30 December 2023–1 January 2024): (**a**) NPSD for signal measurements. (**b**) RSSI power difference of NPSD between rainy days and dry days. (**c**) RSRP power difference of NPSD between rainy days and dry days. (**d**) RSRQ power difference of NPSD between rainy days and dry days. (**e**) SNR power difference of NPSD between rainy days and dry days.

Figure 12 and Figure 13 illustrate the NPSD and the differences between rain events and dry periods in Dataset 1. Two reference dry periods are from 4 December to 6 December and from 3 March to 5 March, as indicated in the legend. For frequencies lower than 0.0417 (×1/3600) Hz, the power of RSSI and SNR measurements during rainy days is higher than that observed on the reference dry day, as depicted in these figures.

**Figure 14:**
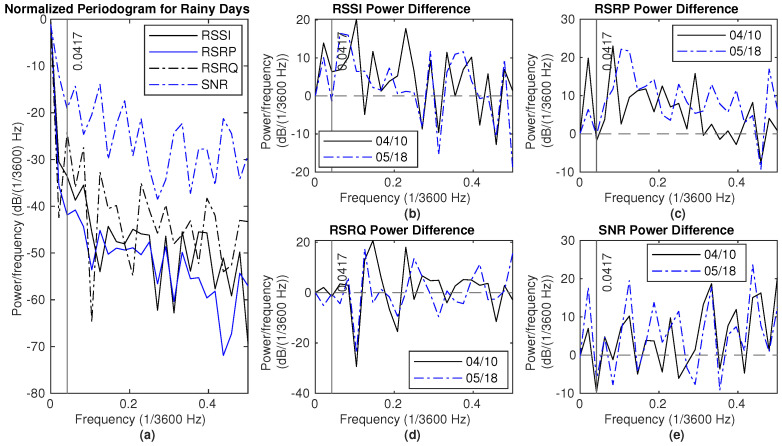
Signal measurements during rain (4–6 April): (**a**) NPSD for signal measurements. (**b**) RSSI power difference of NPSD between rainy days and dry days. (**c**) RSRP power difference of NPSD between rainy days and dry days. (**d**) RSRQ power difference of NPSD between rainy days and dry days. (**e**) SNR power difference of NPSD between rainy days and dry days.

**Figure 15:**
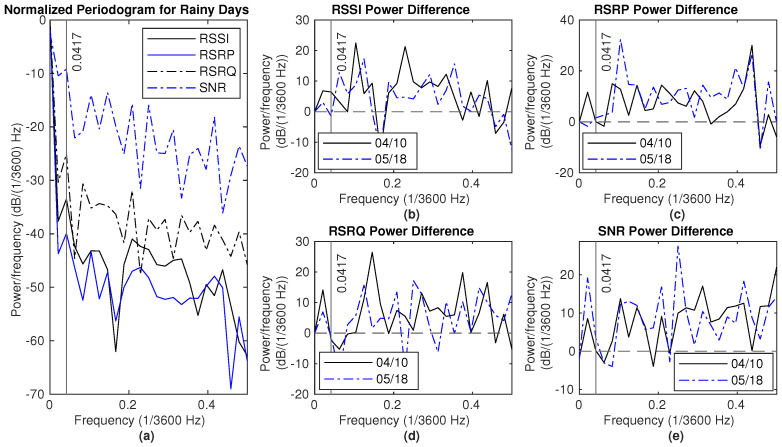
Signal measurements during rain (10–12 May): (**a**) NPSD for signal measurements. (**b**) RSSI power difference of NPSD between rainy days and dry days. (**c**) RSRP power difference of NPSD between rainy days and dry days. (**d**) RSRQ power difference of NPSD between rainy days and dry days. (**e**) SNR power difference of NPSD between rainy days and dry days.

There are two major rain events in Dataset2, from 4 April 2024 to 6 April 2024 and from 10 May 2024 to 11 May 2024. The NPSD and the difference of NPSD between the rainy and the dry period are shown in Figure 14 and Figure 15, respectively. Here, two reference dry periods are selected, from 10 April 12 p.m. to 12 April 12 p.m. and 18 May 12 p.m. to 20 May 12 p.m. The power for RSSI and SNR measurements on rainy days is higher than that of the reference dry day for frequencies below 0.0417 (×1/3600) Hz. Generally, there is more power at lower frequencies, specifically below 0.15 (×1/3600) Hz, in hourly average RSSI measurements, except at 0.0417 (×1/3600) Hz. Additionally, the hourly average RSRP measurement shows greater power in the range of 0.1 to 0.3 (×1/3600) Hz during rain compared to dry conditions.

The power spectral density increase at low frequencies on RSSI and SNR can be attributed to rain-related effects. Firstly, multipath scattering occurs when radio waves reflect off various surfaces such as buildings and the ground, leading to the superposition of multiple signal copies at the receiver. During rainy conditions, the increased atmospheric moisture can further enhance these scattering effects, disrupting the original signal pattern. Secondly, rain-induced attenuation is a critical factor affecting signal propagation. Raindrops absorb and scatter the radio waves, causing a decrease in signal strength. Different propagation paths might have different rain-caused attenuation. RSRP is a measure of the power level of the LTE reference signals, providing a direct indication of the signal strength from the serving cell. RSSI captures the total average signal power, which includes not only the reference symbols but also interference. In NLoS scenarios, the variations in RSRP caused by rainfall can be less pronounced due to the complex nature of microwave propagation and the daily variations observed. Interference is also influenced by rainfall, and the rainfall effects on the reference signal and interference might be different and result in a higher observed power in the low-frequency range of RSSI. RSRQ is an indicator of signal quality, and it can be derived from RSRP and RSSI, as shown in Equation (1). Consequently, the impact of rainfall on RSRQ may be drowned, given that both metrics are intertwined. SNR measures the ratio of received signal strength to noise level. Rainfall can affect both components of the SNR differently, which may lead to a higher proportion of low-frequency components in the signal spectrum.

**Figure 16:**
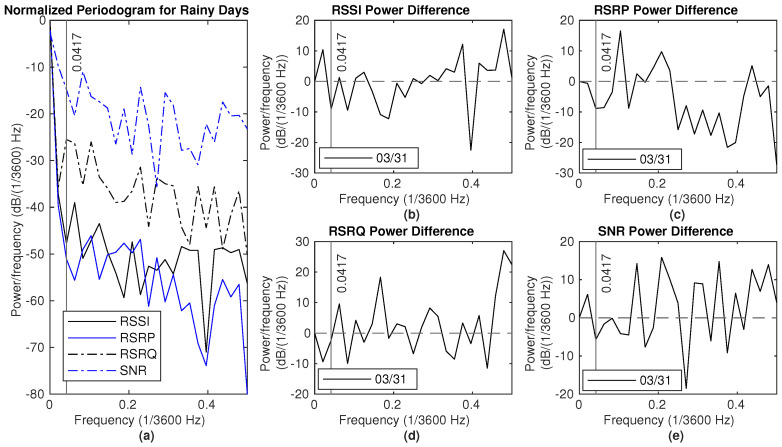
Signal measurements during rain (4–6 April): (**a**) NPSD for signal measurements. (**b**) RSSI power difference of NPSD between rainy days and dry days. (**c**) RSRP power difference of NPSD between rainy days and dry days. (**d**) RSRQ power difference of NPSD between rainy days and dry days. (**e**) SNR power difference of NPSD between rainy days and dry days.

The NPSD and the difference between the rain event and the dry period in Dataset3 are shown in Figure 16. The reference dry days are from 31 March at 12 p.m. to 2 April at 12 p.m. During rainy periods, there is more power in frequencies below 0.0417 (×1/3600) Hz for both RSSI and SNR measurements.

Spectral analysis of the three datasets indicates that rainy days generally exhibit more power at lower frequencies in both RSSI and SNR measurements. Lower frequencies correspond to longer time intervals, and the increased power at these frequencies suggests that signal measurement patterns during rain occur over extended periods compared to dry days. This indicates that rainfall disrupts the signal patterns that typically emerge within shorter time intervals, reinforcing the conclusion that rainfall influences signal measurements.

## 6. Discussion

This section provides an overview of the relevant studies and previous work. Potential future research directions will also be discussed.

Rainfall would cause additional attenuation for radio signal propagation. Many papers studying signal power from CML have observed that the decrease in the signal attenuation and the increase in the precipitation rate matched very well with each other [39,40]. The stability of the measured signal level appears to be influenced by weather conditions, particularly in the context of the 3G LoS scenario [19]. In studies that have collected LTE signal data under the LoS scenario, a discernible difference in the average received signal levels has been noted between rainy and non-rainy conditions [16]. Notably, a significant drop in RSSI has been observed since the beginning of rain [25]. This highlights the sensitivity of signal strength measurements to changes in weather, especially in scenarios where the signal propagation path is not obstructed. However, in our study, where data were collected under the NLoS scenario, such changes in signal measurements associated with rainfall conditions are not apparent. This discrepancy could be attributed to the complexity introduced by obstructions and reflections in the NLoS environment. The presence of multiple signal paths in NLoS conditions could potentially average out the effects of rain, leading to less noticeable fluctuations in signal strength compared to the more direct LoS scenario. Thus, we start with investigating the signal measurements during dry time. A daily signal fluctuation pattern has been observed in this work. Such no-rain-related variations make it challenging to distinguish the signal attenuation increases from rain or the daily pattern itself from the time domain.

One of the biggest differences between CML data and data collected by the smartphone is their propagation environments, which makes the attenuation calculation difficult. Some studies adopt signal measurement during the dry time or previous period as the baseline and normalized the rain signal [16,25]. Such process techniques assume that the transmit power does not change or the received power is not affected by other factors except rainfall over time. In this paper, data collection is conducted in real-life situations, and a daily pattern for the variation of the measured signal strength is found. This makes adopting previous dry time signal measurements as the baseline for calculating the attenuation unsuitable. Thus, we employed the spectral analysis method and found the increased power at lower frequencies in rainy days compared to dry periods.

The diversity of the signal data could provide more information about the influence of rain. For example, CML data usually consist of many links with different frequencies, link distances, and polarization directions. These factors all impact the signal measurement [15]. A rainfall influence on the attenuation when transmitter and receiver are both indoors with NLoS path in self-built 2 GHz horizontal polarization microwave link is studied [38]. Further developed applications with higher sample frequency might speed up the research. Except for the atmospheric effects, network load might also impact the received signal metric. Separation of such an impact would be a direction for future work.

Most existing works are conducted under the GSM/LTE network, which has been gradually replaced by the 5G network. From the open literature, no rainfall prediction study has been conductred based on signal data collected by a user terminal under a commercial 5G network. The possibilities of utilizing sub-6GHz signals with NLoS links as a rainfall monitoring method have been reviewed [41]. By investigating the impact of rainfall on 5G network signal measurements, 5G base stations can be potentially repurposed as opportunistic virtual rain gauges.

## 7. Conclusions and Future Work

This paper studies rainfall impacts on key signal metrics widely available in mobile terminals. The experiments are conducted in real-life scenarios. Both small-scale and large-scale variations in measurements during dry periods are analyzed by comparing them with variations in environmental factors using statistical methods. Our key findings include the identification of a daily pattern in signal strength measurements during dry days, and rainy days tend to show increased power at lower frequencies in signal measurement compared to dry days. These findings have been confirmed by data analysis across various locations and weather conditions. The results indicate novel interesting rainfall impacts on everyday mobile links, potentially facilitating precise rainfall sensing using by-product signals in our mobile devices.

In this work, we mainly exploit the experimental results to reveal novel insights of rainfall’s impacts on cellular signal metrics available in modern mobile terminals. Diverse experimental scenarios are considered to reflect the impacts of different propagation environments, though analytical modeling is not performed yet. Therefore, an interesting future work would be establishing the statistical modeling of various cellular signal metrics under different rainfall conditions as well as diverse channel models.

## Figures and Tables

**Table 2:** Datasets for signal measurements at different locations.

Dataset	Sample Interval (s)	Indoor/Outdoor	Collection Time	Number of Samples
1	5	Indoor	24 October 2023–20 March 2024	1,813,140
2	5	Outdoor	22 March 2024–25 June 2024	798,717
3	30	Indoor	28 March 2024–5 April 2024	20,319

**Table 3:** A snippet of the cellular signal measurements.

PLMN	XCI	xNBID	LOCAL_CID	LAC_TAC	
50,502	20,781,625	81,178	57	52,010	
50,502	20,781,625	81,178	57	52,010	
50,502	20,781,625	81,178	57	52,010	
BAND	RSSI	RSRP_RSCP	RSRQ_ECIO	SNR	LAT
2600 B7	−67	−101	−13	13	−33.886251
2600 B7	−67	−101	−13	13	−33.886251
2600 B7	−67	−101	−13	13	−33.886251
LON	BANDWIDTH	CA	NR_STATE	DATE	TIME
151.195737	20,000	1	none	23 March 2024	10:02:56
151.195737	20,000	1	none	23 March 2024	10:03:01
151.195737	20,000	1	none	23 March 2024	10:03:06

**Table 4:** Main cell information for each dataset.

Dataset	Main Cell ID	Sample Number	Band	Bandwidth (MHz)	Distance to User Terminal (m)
1	20781625	1,339,744	2600 MHz B7	20	195.83
2	20781625	751,792	2600 MHz B7	20	181.08
3	135148290	9450	1800 MHz B3	15	392.27

## Data Availability

The data that support the findings of this study are available from the corresponding author upon reasonable request.
